# Genotypic characterization and antimicrobial resistance profile of *Salmonella* isolated from chicken, pork and the environment at abattoirs and supermarkets in Chongqing, China

**DOI:** 10.1186/s12917-019-2202-4

**Published:** 2019-12-18

**Authors:** Tingting Chen, Jiali Jiang, Chao Ye, Jianhua Xie, Xia Chen, Dongyi Xu, Zheng Zeng, Yuanyi Peng, Dong-Liang Hu, Rendong Fang

**Affiliations:** 1 College of Animal Science and Technology, Southwest University, No. 2 Tiansheng Road, Beibei District, Chongqing, 400715 China; 2 Chongqing Animal Disease Prevention and Control Center; Laboratory of Quality & Safety Risk Assessment for Animal Products on Biohazards, Ministry of Agriculture, Chongqing, 401120 China; 3 Animal Husbandry and Aquatic Products Station of Yubei District, Chongqing, 401120 China; 4 Department of Zoonoses, Kitasato University School of Veterinary Medicine, Towada, 034-8628 Japan

**Keywords:** *Salmonella*, Serotype, Multilocus sequence typing, Antimicrobial resistance, Slaughterhouse, Supermarket

## Abstract

**Background:**

*Salmonella* is one of the most important foodborne pathogens, causing outbreaks of human salmonellosis worldwide. Owing to large scales of consumption markets, pork and poultry that contaminated by *Salmonella* could pose a tremendous threat to public health. The aim of this study was to investigate the contamination of *Salmonella* from chicken, pork and the environment in slaughtering and retail processes in Chongqing, China.

**Results:**

A total of 115 *Salmonella* isolates were recovered from 1112 samples collected from pork, chicken and the environment. Compared with the isolation rate of samples from chicken (9.50%) and the environment (6.23%), samples from pork had a significant higher isolation rate (44.00%). The isolation rates in slaughterhouses (10.76%) and in supermarkets (10.07%) showed no statistical difference. Thirty different serotypes were identified among all the isolates. *S.* Derby (*n* = 26), *S.* London (*n* = 16) and *S.* Rissen (*n* = 12) were the dominant serotypes. Antimicrobial susceptibility testing revealed that 73.04% isolates were resistant to tetracycline, followed by 66.96% to ampicillin and 59.13% to doxycycline. More than half (50.43%) of the isolates were multidrug resistant (MDR), and most of the MDR isolates were from supermarkets. Multilocus sequence typing results showed 24 out of 115 isolates were ST40, which was the most prevalent. Furthermore, isolates from supermarkets had 20 different sequence types while isolates from slaughterhouses only had 8 different sequence types.

**Conclusion:**

Our study highlighted that *Salmonella* was more frequently isolated in pork production chain than that in chicken. Compared with isolates from slaughterhouses, isolates from supermarkets had more MDR profiles and represented a wider range of serotypes and sequence types, indicating that the retail process had more diverse sources of *Salmonella* contamination than that of slaughtering process.

## Background

*Salmonella*, a foodborne pathogen, causes diarrhoeal diseases even death in both humans and animals [1]; it can survive in a dry environment for several weeks or even in water for several months [2]. According to previous surveys, the aetiological agent of salmonellosis largely attributed to contaminated food, which mostly were poultry and pork [3, 4]. In China, pork is the mainstream of meat consumption. Meanwhile, the consumption of poultry is rising year by year. Contamination by *Salmonella* in slaughtering and retail processes of chicken and pork could be a potential pathway to threat public health.

Antibiotics are widely used to improve human and animal health, and also are commonly incorporated into animal feed to improve growth rate and feed efficiency in many countries [5, 6] A previous report showed that China approximately consumed more than 162,000 tons of antibiotics annually, and husbandry industry account for 52.00% of the total consumption. Within the husbandry industry consumed antibiotics, 52.20% antibiotics were in pork production and 19.60% were in chicken production. As for the categories of antibiotic, sulfonamides, tetracyclines, fluoroquinolones, macrolides, β-lactams, and other antibiotics shared 5.00, 7.00, 17.00, 26.00, 21.00, and 24.00% of the total usage [7]. Long-term exposed to antibiotics has led selection pressure to environmental bacterium, which causes antimicrobial resistance and even multidrug resistance (MDR). Antimicrobial resistance in *Salmonella* is a global issue. Large amount of *Salmonellae* resistant to extended-spectrum-β-lactams (ESBLs) and fluoroquinolones, which are important in treating salmonellosis [8]. Drug-resistant *Salmonella*, especially multidrug-resistant *Salmonella,* has been a menace to food safety and human health.

Typing methods used to investigate the characterization of *Salmonella* can help to enrich our knowledge of its regularity of dissemination. Serotyping presents a well-established methodology for typing of *Salmonella* [9]. To date, approximately 2600 serotypes have been discovered. The traditional method for serotyping, the Kauffmann-White-Le Minor Scheme requires a series of antisera, consuming time and money. Hence, a variety of typing methods were established to study the molecular epidemiologic characterization of *Salmonella* with its transmission dynamics, including pulsed-field gel electrophoresis (PFGE), restriction fragment length polymorphism (RFLP), amplified fragment length polymorphism (AFLP), whole-genome sequencing (WGS), and multilocus sequence typing (MLST) [10–13]. Compared with other methods, MLST is a highly repeatable typing method that based on sequence analysis of selected housekeeping genes. Recently, approximately 224,516 *Salmonella* strains has been uploaded by users in the MLST database [14], which becomes a convenient tool for researchers.

Contamination and antimicrobial resistance of *Salmonella* isolated from food-producing animals is severe worldwide [15], particularly in China [16–18]. Furthermore, several studies reported that *Salmonella* isolates could be recovered from farms, slaughterhouses and retail markets [19, 20] and a previous study indicated that *Salmonella* isolates could transmitted from slaughterhouses to retail markets in pig production chain [17]. However, few studies focused on the comparison of *Salmonella* contamination inpork and chicken as well as their slaughtering and retail chains*.* Therefore, the intention of this study was to compare the antimicrobial resistance, and genetic relationship of *Salmonella* isolates recovered from the environment, chicken and pork at abattoirs and supermarkets located in Chongqing, China.

## Results

### Isolation and serotyping of *Salmonella* from samples

A total of 115 *Salmonella* isolates were recovered from 1112 samples collected from slaughterhouses and supermarkets, the isolation rates form pork, chicken and the environment were 44.00, 9.50 and 6.23%, respectively. Samples from pork had significant higher isolation rates than that from chicken and the environment in both slaughterhouses and supermarkets (Table 1). Within the different sources of environmental samples, *Salmonellae* were isolated only from floor, knives and tables at slaughterhouses, but isolated from all the sources of environmental samples at supermarkets, especially chopping boards and ice (Table 2). These results indicated that the environment in supermarkets had more diverse contamination sources than that in slaughterhouses.

**Table 1:** Positive isolation rates of *Salmonella* from different sampling sources

Sampling Site	Sampling Source	No. of Samples	Positive No. of Isolates	Isolation Rate
Slaughterhouse and supermarket	Chicken	242	23	9.50%
	Pork	100	44	44.00%
	Environment	770	48	6.23%
	Total	1112	115	10.34%
Slaughterhouse	Chicken	150	14	9.33%
	Pork	42	18	42.86%
	Environment	245	15	6.12%
	Total	437	47	10.76%
Supermarket	Chicken	92	9	9.78%
	Pork	58	26	44.83%
	Environment	525	33	6.29%
	Total	675	68	10.07%

**Table 2:** Contamination frequency of environmental samples from supermarkets and slaughterhouses

Sampling site	Source	No. of Samples	Positive No. of Isolates	Positive Rate
Slaughterhouse	Wash water	30	0	0
	Knives	30	3	10.00%
	Floor	30	10	33.33%
	Feces	15	0	0
	Apparatus	30	0	0
	Containers	30	0	0
	Tables	30	2	6.67%
	Carcasses	30	0	0
	Blood	20	0	0
	Total	245	15	6.12%
Supermarket	Chopping boards	75	7	9.33%
	Ice	75	6	8.00%
	Knives	75	4	5.33%
	Floor	75	6	8.00%
	Containers	75	4	5.33%
	Wash water	75	3	4.00%
	Tables	75	3	4.00%
	Total	525	33	6.29%

**Fig. 1:**
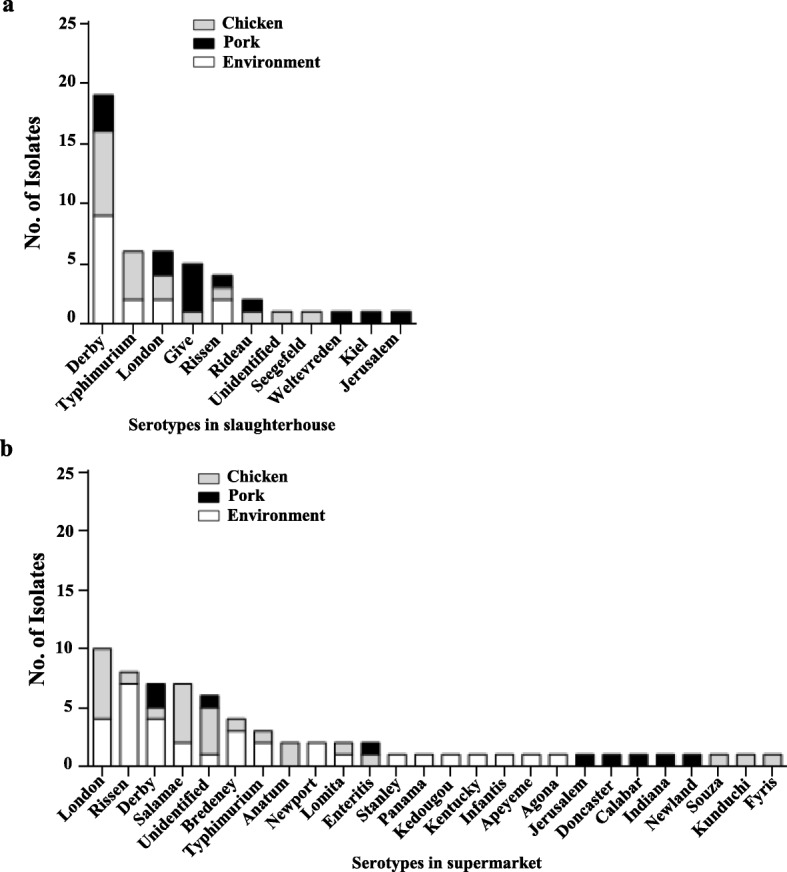
Serotype results of *Salmonella* isolates. **a** Serotype results of isolates from chicken, pork and the environment in slaughterhouses. **b** Serotype results of isolates from chicken, pork and the environment in supermarkets

Thirty serotypes in 108 isolates were successfully identified, except that seven isolates were failed in serotyping. *Salmonella* Derby (*n* = 26), London (*n* = 16), and Rissen (*n* = 12) were the most commonly observed serotypes in this study. Isolates from slaughterhouses had 10 serotypes (Fig. 1a) and supermarkets had 25 serotypes (Fig. 1b). Five serotypes (Derby, Typhimurium, London, Rissen, and Jerusalem) were shared both at supermarkets and slaughterhouses. Five serotypes were only detected at slaughterhouses, while 20 serotypes were only detected at supermarkets. Taken together, isolates from supermarkets showed more diverse serotypes than that from slaughterhouses.

### Antimicrobial susceptibility testing

**Fig. 2:**
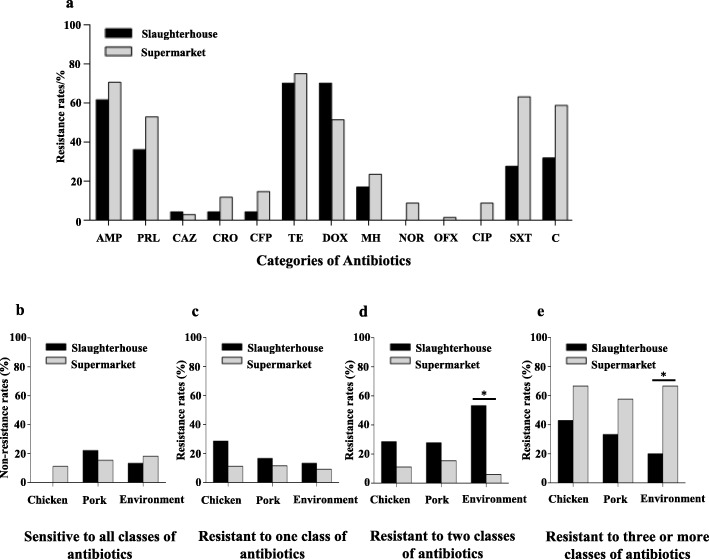
Antimicrobial resistance results of *Salmonella* isolates. **a** The resistance rates of *Salmonella* from different sampling sources. **b** The rates of sensitivity *Salmonella* isolates to all classes of antibiotics. **c** The rates of resistant *Salmonella* isolates to one class antibiotics. **d** The rates of resistant *Salmonella* isolates to two classes antibiotics. **e** The rates of MDR *Salmonella* isolates. Statistical significance was determined by chi-squared test (^*^P < 0.05)

Antimicrobial susceptibility testing of 115 *Salmonella* isolates to 13 antimicrobials was performed. Overall, 85.22% (98/115) isolates were resistant to at least one antibiotic and 50.43% (58/115) were MDR. For samples from both slaughterhouses and supermarkets, isolates showed resistance to tetracycline (73.04%, 84/115) was the highest, followed by 66.96% (77/115) to ampicillin and 59.13% (68/115) to doxycycline (Fig. 2a). The rates of strains that were sensitive or resistant to one or two classes of antibiotics were displayed in Fig. 2b–d. The MDR strains (Fig. 2e) were made up of 25.86% (15/58) at slaughterhouses and 74.14% (43/58) at supermarkets.

### Multilocus sequence typing

**Fig. 3:**
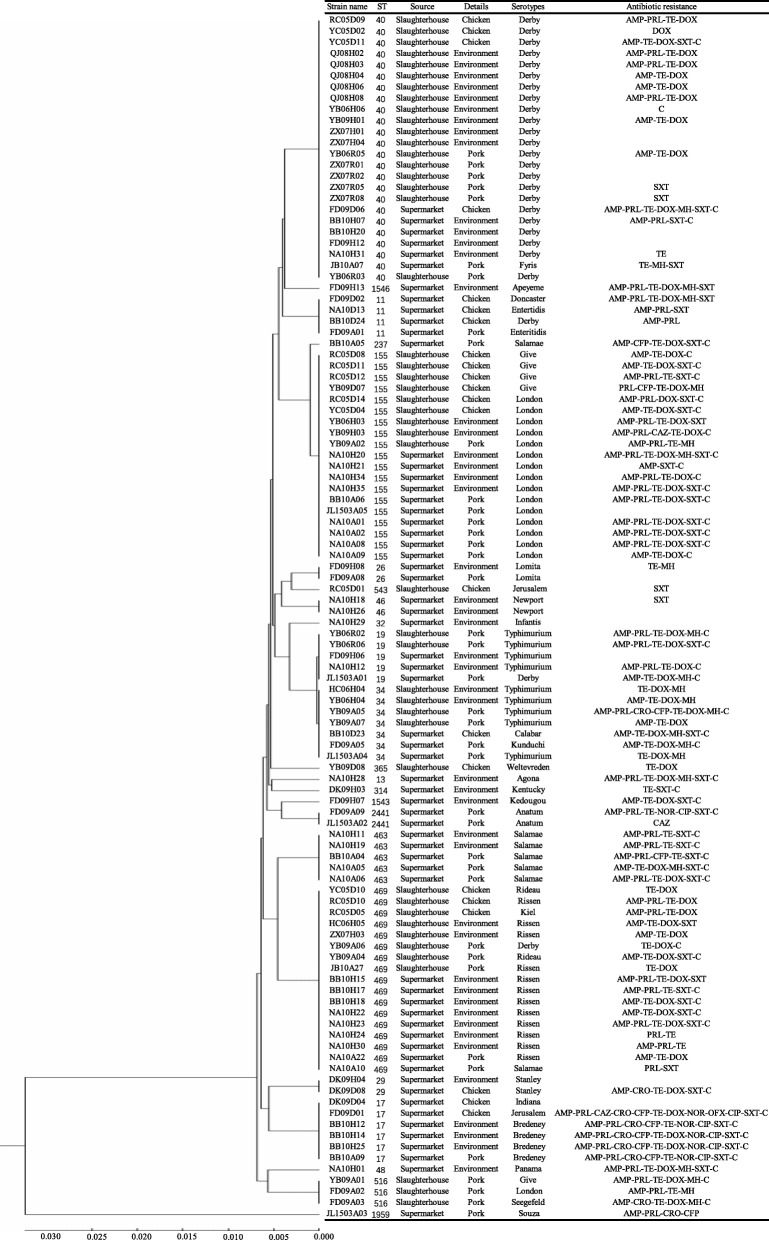
Unweighted pair group method with arithmetic means (UPGMA) dendrogram based on multilocus sequence typing (MLST) profiles of the 108 *Salmonella* isolates from slaughterhouses and supermarkets

Multilocus sequence typing was used to identify the relatedness of *Salmonella* in pork, chicken and the environment from slaughterhouses and supermarkets. Twenty-three different sequence types (STs) were identified among 108 isolates, while 7 isolates with failed serotyping results also defected in MLST were excluded for further analysis (Fig. 3). All sequence types consist of 15 from supermarkets, 3 (ST543, ST365 and ST516) from slaughterhouses and 5 (ST19, ST34, ST40, ST155 and ST469) from both sites. Most of the sequence types were detected in less than 10 isolates, except for ST40, ST155 and ST469. The largest population of isolates were ST40 (*n* = 24), followed by ST155 (*n* = 19) and ST469 (*n* = 17). Some *Salmonella* isolates presented similar sequence types that belonged to the same serovar. For example, all ST40 isolates were *S*. Derby, all *S*. Rissen isolates belonged to a single cluster (ST469) and 15 out of 16 *S.* London isolates were ST155. As for the relationship between MLST and antibiotic resistance, we found that ST17 and ST155 were resistant to a wide range of antibiotics in this study, especially that 16 out of 18 ST155 isolates were MDR strains (Fig. 3).

## Discussion

For the purpose of this study, *Salmonella* isolates were recovered from scores of sites, including chicken, pork and the environment at abattoirs and supermarkets. Our results indicated that *Salmonella* was more frequently isolated in pork, and supermarkets exhibited a higher MDR *Salmonella* isolation rate and more diversity in serotypes and sequence types than slaughterhouses.

The overall isolation rate of *Salmonella* in our study was 10.25%, which was lower than previous studies conducted in Sichuan province [11] and Yangzhou city [17], but close to surveys in three provinces of central China [21] and Germany [22]. It should be noted that although isolation rate at slaughterhouses (10.76%) was similar to that at supermarkets (10.07%), *Salmonella* appeared more frequently in pork (44.00%) than that in chicken (9.50%). Other studies also showed that *Salmonella* contamination rates in pork varies from 30 to 70% at retail markets [23, 24] and from 10 to 50% at slaughterhouses [25, 26], indicating that poor control measures were performed in slaughtering and retail chains of pork. For example, poor general hygiene and unsuitable storage conditions were commonly detected in pig slaughterhouses. Also, lacking of appropriate storage methods and regular disinfection increased the risk of *Salmonella*-colonizing activity at retail markets. In general, all of the differences attributed to collection seasons, amounts of samples and types, isolation methods and management.

*S.* Derby, generally detected in pork, could cause salmonellosis in many countries [27], and *S.* Rissen was generally considered to be transported through pig products in European countries [28]. In this study, *S.* Derby and *S.* Rissen were isolated from pork, chicken and the environment, and most of *S.* Derby isolates were detected in the environment. *S.* Derby was the dominant serotype, which was similar to other studies [11, 17]. *S*. Typhimurium and *S.* Enteritidis are the main serotypes causing acute human infection [29], and in this study 11 isolates were detected to be *S.* Enteritidis or *S.* Typhimurium, which had potential threats to public health. In addition, more categories of serotypes and sequence types were detected in isolates from supermarkets than that from slaughterhouses, demonstrating the various and abundant sources of contamination in retail process. Some STs in this study related to specific serovars, for instance *S.* London with ST155, ST469 with *S.* Rissen, and ST40 with *S.* Derby. Our results supported the conjecture that multilocus sequence typing could be an alternative method for serotyping in the future [30].

In this study, most of the isolates showed resistance to tetracycline and ampicillin, which was similar to a previous study [31]. The high resistance rate to these two antibiotics is reasonable, since these two antibiotics has been largely used in the husbandry industry in China [7]. It was noteworthy that more than half of the isolates exhibited MDR profiles, and compared with slaughterhouses, MDR *Salmonella* contamination occurred at supermarkets was much more frequently. The sanitation control strategies in supermarkets need to be carried out in this area to improve the safety of animal products.

## Conclusion

In summary, the results of this study indicated that the contamination of *Salmonella* occurred in pork made it become a potential reservoir for human infection to some extent. In addition, although the isolation rate at supermarkets was close to that at slaughterhouses, isolates from supermarkets presented a high frequency of MDR profiles and a wider range of serotypes and sequence types; these results indicated that *Salmonella* isolates from supermarkets were more threatening and their sources were much more complicated than that from slaughterhouses. Therefore, strict hygiene method and HACCP management in retail process should be taken into consideration to prevent foodborne infection caused by *Salmonella*.

## Methods

### Sample collection

Convenience sampling was carried out in 7 slaughterhouses and 5 supermarkets in 12 districts of Chongqing, China. From March to October in 2015, a total of 1112 samples were isolated. Chicken and pork were unpacked fresh meat; slaughtering environment including wash water (*n* = 30), knives (n = 30), floor (n = 30), feces (*n* = 15), apparatus (n = 30), containers (n = 30), tables (n = 30), carcasses (n = 30), and blood (*n* = 20); retail environment including chopping boards (*n* = 75), ice (n = 75), knives (n = 75), floor (n = 75), containers (n = 75), wash water (n = 75), and tables (n = 75). All collected samples were stored in an icebox and transported to a laboratory within 2 h of collection for immediate processing and then held in a refrigerator at 4 °C.

### Isolation and serotyping

After a pre-enrichment step of each sample in 10 mL sterile buffered peptone water (BPW) and incubated overnight at 37 °C. 0.2 mL of each pre-enriched suspensions were added into 2 ml of Rappaport-Vassiliadis enrichment Broth (RVB) and 2 ml of Tetrathionate broth (TTB) respectively, then incubated at 42 °C for 24 h. One loopful of each RVB and TTB culture was then streaked onto Xylose Lysine Tergitol 4 (XLT-4) agar plates, which were incubated at 37 °C for 24 to 48 h. Among suspected colonies, only one was picked up from a plate and confirmed by specific gene through Polymerase Chain Reaction (PCR) of *Salmonella* using assays. Each isolate was serotyped by slide agglutination based on the Kauffmann-White-Le Minor Scheme [32].

### Antimicrobial susceptibility testing

The standard Kirby-Bauer disk diffusion method recommended by the Clinical and Laboratory Standards Institute (CLSI, 2010) was carried out to test antimicrobial susceptibility of the *Salmonella* isolates to 13 categories of antimicrobials (Hangzhou Microbial Reagent., Ltd.): ampicillin (AMP 10 μg), cefoperazone (CFP 75 μg), piperacillin (PRL 100 μg), tetracycline (TE 30 μg), ceftazidime (CAZ 30 μg), doxycycline (DOX 30 μg), ceftriaxone (CRO 30 μg), minocycline (MH 30 μg), norfloxacin (NOR 10 μg), sulfamethoxazole (SXT 1.25 μg), ofloxacin (OFX 5 μg), chloramphenicol (C 30 μg) and ciprofloxacin (CIP 5 μg). *Escherichia coli* ATCC 25922 was invoked as the control organism. According to the CLSI, the isolates were considered to be susceptible, intermediate, or resistant. *Salmonella* isolates resistant to three or more antimicrobial classes were defined as MDR isolates.

### Multilocus sequence typing

Protocols used for MLST of *Salmonella* were described online [33]. Seven housekeeping genes were amplified by PCR, including *thrA, purE, sucA, hisD, aroC, hemD, and dnaN.* PCR products were purified and sequenced by Sanger method, and the alleles and STs were assigned according to the MLST scheme [34]. The unweighted pair group method with arithmetic means analysis (UPGMA) was utilized to infer relationships among the isolates through MEGA7 software [35].

### Statistical analysis

All statistical analyses were done using SPSS 20.0 (SPSS Inc., Chicago, IL), and the chi-squared test was applied to assess any statistically significant (*P* < 0.05) differences in this study.

## Data Availability

The datasets used and/or analyzed during the current study available from the corresponding author on reasonable request.

## Abbreviations

AMP: ampicillin
C: chloramphenicol
CAZ: ceftazidime
CFP: cefoperazone
CIP: ciprofloxacin
CLSI: Clinical and Laboratory Standards Institute
CRO: ceftriaxone
DOX: doxycycline
ESBL: Extended-spectrum-β-lactams
MDR: multidrug resistance
MH: minocycline
MLST: multilocus sequence typing
NOR: norfloxacin
OFX: ofloxacin
PRL: piperacillin
ST: sequence type
SXT: sulfamethoxazole
TE: tetracycline
UPGMA: the unweighted pair group method with arithmetic means analysis
